# Comparison of Ethnic-specific Databases in Heidelberg Retina Tomography-3 to Discriminate Between Early Glaucoma and Normal Chinese Eyes

**DOI:** 10.2174/1874364101711010040

**Published:** 2017-02-28

**Authors:** Xiu Ling Tan, Sae Cheong Yap, Xiang Li, Leonard W. Yip

**Affiliations:** 1 Department of Ophthalmology, Tan Tock Seng Hospital, National Healthcare Group Eye Institute, Singapore; 2 Ministry of Health Holdings, Singapore; 3 Department of Statistics and Applied Probability, National University of Singapore, Singapore

**Keywords:** Glaucoma diagnosis, glaucoma imaging, tomography, ethnicity, sensitivity, specificity

## Abstract

**Purpose:**

To compare the diagnostic accuracy of the 3 race-specific normative databases in Heidelberg Retina Tomography (HRT)-3, in differentiating between early glaucomatous and healthy normal Chinese eyes.

**Method:**

52 healthy volunteers and 25 glaucoma patients were recruited for this prospective cross-sectional study. All underwent standardized interviews, ophthalmic examination, perimetry and HRT optic disc imaging. Area under the curve (AUC) receiver operating characteristics, sensitivity and specificity were derived to assess the discriminating abilities of the 3 normative databases, for both Moorfields Regression Analysis (MRA) and Glaucoma Probability Score (GPS).

**Results:**

A significantly higher percentage (65%) of patients were classified as “within normal limits” using the MRA-Indian database, as compared to the MRA-Caucasian and MRA-African-American databases. However, for GPS, this was observed using the African-American database. For MRA, the highest sensitivity was obtained with both Caucasian and African-American databases (68%), while the highest specificity was from the Indian database (94%). The AUC for discrimination between glaucomatous and normal eyes by MRA-Caucasian, MRA-African-American and MRA-Indian databases were 0.77 (95% CI, 0.67-0.88), 0.79 (0.69-0.89) and 0.73 (0.63-0.84) respectively. For GPS, the highest sensitivity was obtained using either Caucasian or Indian databases (68%). The highest specificity was seen with the African-American database (98%). The AUC for GPS-Caucasian, GPS-African-American and GPS-Indian databases were 0.76 (95% CI, 0.66-0.87), 0.77 (0.67-0.87) and 0.76 (0.66-0.87) respectively.

**Conclusion:**

Comparison of the 3 ethnic databases did not reveal significant differences to differentiate early glaucomatous from normal Chinese eyes.

## INTRODUCTION

Glaucoma is a progressive optic neuropathy with characteristic optic disc changes and corresponding visual field abnormalities. As structural changes in the glaucomatous optic discs often precede functional changes (*e.g.* perimetric visual field defects), clinicians have incorporated various adjunctive instruments and imaging tools in routine clinic practice to aid detection of early glaucoma.

The Heidelberg Retina Tomograph (HRT; Heidelberg Engineering, Heidelberg, Germany) is a Confocal Scanning Laser Ophthalmoscope (CSLO) used frequently as an optic disc imaging tool. Earlier versions of the HRT analysis programs include linear discriminant functions, such as FS Mikelberg discriminant function (FSM) and RB discriminant function (RB), while the HRT-II software had the Moorfields Regression Analysis (MRA). However, this requires an operator to manually draw an outline of the optic disc. Another disadvantage was that the HRT-II normative database was from 112 subjects of European ancestry, thus limiting its application to other populations. For example, it has been shown that Moorfields Regression Analysis (MRA) has poorer diagnostic ability in subjects with large discs, such as African-Americans [1]. These issues were addressed in the newer version HRT-III software upgrade, which included a novel optic disc analysis algorithm that automatically provides a Glaucoma Probability Score (GPS). The normative database was also expanded to consist of 733 Caucasians, 215 Africans, and approximately 100 Asian Indians [2].

The effect of this expanded normative data into the HRT-III software has been studied. A study by Zelefsky *et al.* [3] found that the HRT-III database increased the sensitivities for both Caucasian and African-American subjects. The specificities were maintained for Caucasians, but decreased for the African-American group. However, it has not been studied in an Asian Chinese population, in particular with regard to the suitability of the 3 ethnic-specific databases.

Studies have reported morphological differences in the optic nerve head of Chinese subjects, compared to other populations. For example, the Handan Eye Study found that the rural Chinese populations had larger optic disc areas, as compared to the Caucasian and Japanese populations. This observation was also evident in the Indian and urban Chinese populations [4]. In view of the estimated 15.8 million Chinese with glaucoma in 2010 and a projected increase to 21.8 million by 2020 [5], it is needful to know which ethnicity database to apply, especially in the absence of a Chinese-specific database. The aim of our study is to compare the performance of the 3 race-specific normative Heidelberg Retina Tomography (HRT)-III databases (Caucasian, African-American and Indian) in the discrimination of healthy and glaucomatous Chinese eyes.

## MATERIALS AND METHODOLOGY

In this prospective cross-sectional study, we recruited 77 Chinese subjects. All were ≥21 years with best-corrected visual acuity (BCVA) of 6/12 or better, and spherical refraction not exceeding 6 dioptres sphere and 3 dioptres cylinder. Exclusion criteria included ocular pathology, amblyopia, significant media opacities with poor fundal view, or other systemic disease (*e.g.* neurological conditions) that can affect visual fields. All subjects completed a standardized interview, detailed ophthalmic examination, standard automated perimetry and HRT-III optic disc imaging. The study was approved by the local ethics committee and conducted in accordance with the Declaration of Helsinki. Informed consent was obtained from all patients prior to recruitment.

The subjects were classified into 2 groups: healthy normal subjects and early glaucoma patients. Normal subjects had intraocular pressures of less than or equal to 21 mm Hg, normal 24-2 Humphrey Visual Fields (HVFs) and did not have a history of glaucoma. To minimise bias, the optic disc appearances were disregarded. Early glaucoma was diagnosed if visual field defects were noticeable on 2 consecutive HVFs, with the Mean Deviation (MD) smaller than -6.0 decibels (dB). The visual field inclusion criterion indicative of glaucomatous damage [6] was the presence of (a) 3 adjacent points decreased by 5 dB from normal age values, with one of these points reduced by at least 10 dB, (b) 2 adjacent points decreased by 10 dB, or (c) 3 adjacent points just above or below the nasal horizontal meridian decreased by 10 dB. None of these points were allowed to be edge points, except for those immediately above or below the horizontal meridian. The HVF was considered to be reliable if there were <30% fixation losses, false-positive responses and false-negative responses. Reliable visual fields were used for analysis. One eye from each normal patient was randomly selected. If the patient had glaucoma in only one eye, that eye was used for analysis. If both eyes had glaucoma, one of the two eyes was randomly chosen.

Topographic optic nerve head imaging was performed with the HRT machine in all subjects. A single operator was responsible for manually drawing the optic nerve head margin and acquiring the images. Only images with a standard deviation (SD) of less than 50 um were included. The data were subsequently exported to the HRT-III software for further analysis. The HRT-III outputs derived were the conventional stereometric parameters, MRA set to the Caucasian database (denoted as MRA3C, C for Caucasian) and GPS set to the Caucasian database (denoted as GPS3C, C for Caucasian). The MRA and GPS were analyzed the second time with the ethnicity database set to African-Americans (MRA3-A, GPS3-A), and the third time with the ethnicity database set to Indians (MRA3-I, GPS3-I). Both MRA and GPS results yielded 3 possible outcomes: within normal limits (WNL), borderline (BL), or outside normal limits (ONL).

Statistical analysis was performed using R version 2.13.2 (R Development Core Team, 2011). Demographics of the study population were reported using proportions/means with standard deviation (SD). Differences between the 2 subject groups were assessed using Wilcoxon Rank Sum test or Chi-square test, wherever appropriate. We evaluated the percentage of eyes classified into WNL, BL, and ONL by each of the 3 ethnic databases. Multinomial logistic regression with cluster was performed to adjust for confounders of age, gender and optic disc area, for glaucoma prediction by MRA and GPS. Sensitivity and specificity were calculated to assess the performances of three individual normative databases, for both MRA and GPS. Area under receiver operating characteristics curve (AUC) was used to evaluate the abilities of each database in discriminating healthy from glaucomatous eyes. AUC of 1.0 indicates perfect discrimination, while AUC of 0.5 shows chance discrimination. The most specific criteria for AUC, sensitivity and specificity were calculated with borderline results of MRA and GPS considered as WNL. The least specific criteria, on the other hand, were derived with borderline results considered as ONL. In addition, Cohen’s Kappa was used for evaluation of concordance between the various analysis methods and databases.

## RESULTS

77 eyes were analysed (52 normal, 25 glaucoma) Table **1** shows the characteristics of the study population. Normal subjects were statistically significantly younger than glaucoma subjects (p<0. 001). Glaucoma patients had a smaller rim-disc area ratio (p<0.001) and thinner retinal nerve fibre layer (p=0.004), compared to the normal subjects. The visual field mean deviation and pattern standard deviation of glaucoma patients were -5.10 ± 0.75 decibels and 6.04 ± 1.73 decibels respectively.

Table **2** summarises the MRA and GPS results using the 3 ethnic-specific databases in the HRT-III software. For the MRA results, 65% of patients were classified as “within normal limits” using the Indian database, as compared to 56% in Caucasian and 60% in African-American databases. This was statistically significant (p=0.004), after adjusting for age and gender. Disc area was not a confounder for MRA (p=0.255). For GPS, 65% of patients were reported as “within normal limits” using the African-American database, as compared to 39% in both Caucasian and Indian databases. This was statistically significant (p<0.001) after adjusting for age, gender and optic disc area (p<0.001).

The sensitivity, specificity, AUC values for the various analysis methods are shown in Table **3**. With borderline results considered as normal (most specific criteria), the highest sensitivity for MRA was obtained with the Caucasian (MRA3-C) and African-American (MRA3-A) databases (68%), while the highest specificity was seen using the Indian (MRA3-I) database (94%). With borderline results considered as abnormal (least specific criteria), the highest sensitivity was seen only in MRA3-C (88%), while the highest specificity was from MRA3-I (85%). With borderline results considered as normal, the Caucasian and Indian databases for GPS had the highest sensitivity of 68%. The highest specificity was seen with the African-American database (98%). With borderline results considered as abnormal, the trend was similar with a sensitivity of 88% and specificity of 83%.

Table **4** shows the kappa coefficient to evaluate the agreement between the various analysis methods and ethnicity databases. There was good agreement (κ = 0.748, p<0.001) among the 3 ethnic-specific databases for MRA (MRA3-C *vs.* MRA3-A *vs.* MRA3-I). There was moderate agreement (κ = 0.578, p<0.001) for GPS (GPS3-C *vs.* GPS3-A *vs.* GPS3-I). Comparing MRA and GPS within each specific ethnicity database, there was fair agreement.

## DISCUSSION

It is a clinical challenge to diagnose early glaucoma. Various statistical formulae have been created to discriminate normal from early glaucomatous eyes. However, it has been neglected that in populations without a correct ethnicity normative database, the machine default choice to the Caucasian normative database may not be appropriate. Hence, our study was designed to compare the diagnostic ability of each of the 3 ethnic-specific normative databases in HRT-III to differentiate normal and early glaucoma patients, so as to evaluate which database would be the most suitable in Chinese eyes.

The first finding was that the MRA and GPS results were affected by the choice of normative database. A higher percentage of patients were reported as “within normal limits” using the African-American database for GPS and the Indian database for MRA, and this was statistically significant. A possible explanation is that GPS provides only a probability value of the likelihood of glaucoma [7]. Reports from ADAGES [8] have described larger optic disc areas and smaller rim-to-disc area ratios in African-Americans compared to Caucasians. De Leon-Ortego *et al.* [9] reported that GPS provided incorrect classifications related to optic disc size. Glaucomatous eyes inaccurately classified by GPS were found to have smaller mean disc areas, while control eyes inaccurately classified had larger disc areas. Hence, applying the African-American normative database to our cohort may classify a higher percentage of patients as “within normal limits”. It is unclear why the MRA-Indian database classified more patients as “within normal limits”.

Another finding was that no single normative database yielded both the highest sensitivity and specificity for MRA and GPS. The current literature suggests that GPS has a higher sensitivity than MRA. Harizman *et al.* [10] conducted a prospective study comparing MRA and GPS to differentiate glaucomatous and normal eyes using HRT-III with race- adjusted ethnicity databases. They reported sensitivities of 71.4% for MRA and 77.1% for GPS, and specificities of 91.9% for MRA and 90.3% for GPS. The trends of a higher sensitivity for GPS and a higher specificity for MRA were also echoed in Yip *et al.* [11]. However, this was only evident with the Indian database in our study. On the other hand, Saito *et al.* [12] reported a higher specificity and lower sensitivity with MRA, as compared to GPS.

Another factor that affects the sensitivity and specificity is the severity of glaucoma. Ferreras *et al.* [13] reported lower sensitivities of both algorithms in early glaucomatous eyes, especially for MRA [10]. Higher diagnostic accuracies were seen in cases of advanced visual loss and glaucoma severity, for both MRA and GPS [14].

Although there was good agreement amongst the 3 ethnic-specific MRA databases, the agreement between the classifications by GPS *versus* MRA within each individual ethnic database was lower. The reason is due to the difference between the 2 formulae. MRA requires a operator to manually outline the optic disc margin, after which the measured and predicted rims areas are compared to classify eyes as outside normal limits, borderline, or within normal limits. On the other hand, GPS analysis is operator-independent and does not require a contour line or reference plane. The images of the optic nerve head are captured and relevant parameters (such as cup size, cup depth, neuroretinal rim steepness, horizontal and vertical retinal nerve fibre layer curvatures) are extracted. These data are then analysed via a Bayer classifier to estimate the probability of glaucomatous damage.

Consequences of the indiscriminate application of a single ethnic database can be understood by reviewing the results of published studies. Direct population comparison is difficult as majority of the studies are done in Caucasian populations [13, 15-18]. Coops *et al.* [15] studied 95 Caucasian healthy controls (mean deviation of -0.1 decibels, range +2.5 to -3.7) and 121 Caucasian glaucoma patients (mean deviation of -3.6 decibels, range +2.0 to -9.9). The results revealed a sensitivity of 56% and specificity of 87% with MRA, and a sensitivity of 59% and specificity of 91% with GPS. One unique study, which applied a Caucasian normative database to a non-Caucasian population, was the Tajima study [12]. Saito *et al.* evaluated the specificities and sensitivities of MRA and GPS in a Japanese population setting of 2182 normal subjects, 49 glaucoma suspects (mean deviation = -1.73±5.53 decibels) and 66 patients with definite glaucoma (mean deviation = -4.90±5.50 decibels). They described a lower sensitivity of 39.4% and higher specificity of 96.1% with MRA, and a higher sensitivity of 65.2% and lower specificity of 83.0% with GPS. This indicates that there may be an important role for an appropriate ethnicity database for each patient, where available.

Apart from CSLO, various imaging techniques such as Spectral-domain Optical Coherence Tomography (SDOCT) have been employed to evaluate the impact of racial differences on the ability of glaucoma detection. Girkin *et al.* [19] reported several race-specific parameters measured with CSLO (*e.g.* cup shape, contour line modulation) that were independently linked to early glaucoma. These parameters contrasted greatly between African Americans and Whites, despite taking into account differences in optic disc areas. On the other hand, Knight *et al.* [20] utilized SDOCT to measure optic nerve head parameters among European, Chinese, Hispanic and African normal subjects. They found that there were small significant dissimilarities in optic nerve head parameters and RNFL thickness across ethnic groups, after adjusting for age. However, after a linear adjustment for disc area, these differences were no longer statistically significant. Likewise, a later study published by Girkin *et al.* [21] in African and European subjects found that that race did not modify the diagnostic interpretation of SDOCT to detect glaucoma.

This puts forward the question if there is a real clinical necessity for ethnic-specific normative databases. In our study, we found that the AUC values for discrimination between early glaucomatous and normal eyes were slightly higher for both MRA and GPS using the African-American normative database. However, our study had a small sample size and was not powered to detect any significant differences between the 3 databases. We feel that this trend should be further evaluated and an adequately powered study may demonstrate the benefits of an ethnicity-specific database. Currently, the need for an ethnic-specific database still remains controversial.

Our study is the first study to compare the normative HRT-III databases with one another, to our knowledge. Standardized protocols were followed to collect patients’ data and optic nerve head parameters. We tried to minimise operator bias by having only one technician draw the contour lines. However, there are a few limitations too. The sample size of our study is small. The glaucoma patients do not have age-matched normal controls and there is a wide age gap between the normal and glaucoma subjects. We recognise that normal ageing itself may also cause changes in optic nerve head characteristics. Several population-based studies such as the Rotterdam Study [22] and the Baltimore Eye Survey [23] showed that age was not associated with disc area, while the Tajimi Study [24] and Handan Eye Study [4] reported otherwise. We attempted to minimise age as a confounder bias by adjusting for it in our statistical analyses.

In conclusion, comparison of the current 3 ethnic databases did not yield significant differences to differentiate between early glaucomatous and normal Chinese eyes.

## Figures and Tables

**Table 1:** Demographics of study population.

Characteristics	Normal Subjects (N = 52)	Glaucoma Subjects (N = 25)	P value
Age (years)	38.2 ± 13.7	62.0 ± 9.6	<0.001*
Gender			
Male	18 (34.6%)	13 (52%)	0.145^†^
Female	34 (65.4%)	12 (48%)	
Optic disc area	2.08 ± 0.39	2.39 ± 0.77	0.267*
Rim disc area ratio	0.75 ± 0.13	0.53 ± 0.19	<0.001*
RNFL thickness	0.26 ± 0.08	0.16 ± 0.18	0.004*
Visual field mean deviation, dB	-0.64 ± 0.65	-5.10 ± 0.75	<0.001*
Visual field pattern standard deviation, dB	1.72 ± 0.44	6.04 ± 1.73	<0.001*

*Wilcoxon Rank Sum test, †Chi-square test.

**Table 2:** Moorfields regression analysis (MRA) and glaucoma probability score (GPS) results using heidelberg retina tomography (HRT)-III.

	HRT III Ethnic-specific Databases			
Analysis Method	Caucasian	African-American	Indian	P value*
MRA (N = 77)				0.004
Within normal limits	43 (56%)	46 (60%)	50 (65%)	
Borderline	10 (13%)	9 (11%)	11 (14%)	
Outside normal limits	24 (31%)	22 (29%)	16 (21%)	
GPS (N =77)				<0.001
Within normal limits	30 (39%)	50 (65%)	30 (39%)	
Borderline	22 (29%)	12 (16%)	22 (29%)	
Outside normal limits	25 (32%)	15 (19%)	25 (32%)	

*Multinomial logistic regression with cluster.

**Table 3:** Sensitivity and specificity values of various analysis methods and databases compared with clinical diagnosis.

Ethnic-specific Database	With Borderline Considered as Normal				With Borderline Considered as Abnormal		
	AUC (95% CI)	Sensitivity (%)	Specificity (%)		AUC (95% CI)	Sensitivity (%)	Specificity (%)
MRA							
Caucasian	0.77 (0.67, 0.88)	68	87		0.83 (0.74, 0.91)	88	77
African- American	0.79 (0.69, 0.89)	68	90		0.79 (0.70, 0.89)	80	79
Indian	0.73 (0.63, 0.84)	52	94		0.80 (0.70, 0.90)	76	85
GPS							
Caucasian	0.76 (0.66, 0.87)	68	85		0.70 (0.61, 0.79)	88	52
African- American	0.77 (0.67, 0.87)	56	98		0.77 (0.67, 0.88)	72	83
Indian	0.76 (0.66, 0.87)	68	85		0.70 (0.61, 0.79)	88	52

MRA: Moorfields Regression Analysis; GPS: Glaucoma Probability Score; AUC: Area Under Curve.

**Table 4:** Agreement between the analysis methods and ethnicity-specific databases.

Analysis Methods and Databases	κ Coefficient	P Value
MRA3-C *vs.* MRA3-A *vs.* MRA3-I	0.748	<0.001
GPS3-C *vs.* GPS3-A *vs.* GPS3-I	0.578	<0.001
MRA3-C *vs.* GPS3-C	0.382	<0.001
MRA3-A *vs.* GPS3-A	0.369	<0.001
MRA3-I *vs.* GPS3-I	0.365	<0.001

C: Caucasian; A: African-American; I: Indian, MRA: Moorfields Regression Analysis, GPS: Glaucoma Probability Score.
